# The Health Effects of Low Glycemic Index and Low Glycemic Load Interventions on Prediabetes and Type 2 Diabetes Mellitus: A Literature Review of RCTs

**DOI:** 10.3390/nu15245060

**Published:** 2023-12-10

**Authors:** Maria Peres, Helena S. Costa, Mafalda Alexandra Silva, Tânia Gonçalves Albuquerque

**Affiliations:** 1 Research and Development Unit, Department of Food and Nutrition, National Institute of Health Dr. Ricardo Jorge, Avenida Padre Cruz, 1649-016 Lisbon, Portugal; maria.peres@insa.min-saude.pt (M.P.); mafalda.silva@insa.min-saude.pt (M.A.S.); tania.albuquerque@insa.min-saude.pt (T.G.A.); 2 REQUIMTE-LAQV, Faculty of Pharmacy, University of Oporto, Rua Jorge Viterbo Ferreira, 228, 4050-313 Porto, Portugal

**Keywords:** glycemic index, glycemic load, prediabetes, type 2 diabetes mellitus

## Abstract

Diets with a low glycemic index (GI) and a low glycemic load (GL) can improve glycemic control, blood lipids, blood pressure and BMI in prediabetes and type 2 diabetes (T2DM), but evidence regarding other aspects of cardiometabolic health is limited. We searched the literature for RCTs published from 2013 to 2023 and reviewed the evidence on low-GI/GL diets and their effects on different aspects of health in prediabetes and T2DM, aiming to build a report on all relevant outcomes included in the studies. We included 14 RCTs with 1055 participants, who were mostly middle-aged individuals with T2DM. Interventions were mostly low GI and lasted 1–36 months. Low-GI/GL foods and diets showed benefits in terms of short-term glycemic control, weight and adiposity. Longer-term trials would be necessary to determine whether these benefits persist over time and/or lead to lower CVD risk and mortality. Effects on lipid profile were inconsistent. Some studies also reported positive effects of low-GI/GL interventions on blood pressure, inflammatory biomarkers, renal function and gut microbiota composition. Future trials should focus on some of these novel outcome measures, which may provide important insights into the metabolic effects of low-GI diets on individuals with diabetes.

## 1. Introduction

Type 2 diabetes mellitus (T2DM) is a chronic disease characterized by a state of hyperglycemia, which derives from impaired insulin secretion by pancreatic islet β-cells and insulin resistance [1]. T2DM represents a major burden to public health, affecting 437.9 million people around the globe with increasing prevalence, especially in developing countries [2]. T2DM is the ninth leading cause of death and the seventh leading cause of disability-adjusted life years (DALYs) [3], which is a measure of loss of healthy years of life [4]. Most individuals with T2DM develop some type of complication throughout the course of the disease [5], with cardiovascular disease (CVD) representing the main cause of morbidity and mortality in this population [6,7].

Prediabetes is a condition that precedes T2DM, in which hyperglycemia does not yet meet the threshold for a diabetes diagnosis [8]. Individuals with prediabetes have a high risk of developing diabetes and often exhibit other cardiometabolic risk factors, such as high blood pressure and dyslipidemia [9].

Several risk factors for T2DM are modifiable and are direct or indirectly linked to unhealthy lifestyle and dietary habits [10]. A strong body of evidence suggests that adopting a healthy lifestyle reduces long-term risk of developing T2DM and delays its onset [11,12,13,14], and may also reduce the associated CVD and mortality risk [12,14,15].

Dietary habits and lifestyle are crucial aspects of T2DM prevention and management [16,17]. Different nutritional approaches may be used, such as diets with a low glycemic index (GI) and a low glycemic load (GL). The GI is a concept introduced by Jenkins et al. in 1981 [18] to measure the quality of carbohydrates and to classify carbohydrate-rich foods, according to their effect on postprandial glycaemia. The GI of a food represents its ability to increase blood glucose compared to that of a reference food—usually glucose or white bread, which have an attributed GI value of 100 [19,20]. Foods containing carbohydrates can be categorized as low (≤55), medium (56–69), or high GI (≥70) [21]. This means that high-GI foods induce a bigger and faster rise in blood glucose, since the carbohydrates they contain are digested and absorbed more quickly than in low-GI foods [20]. 

The concept of GL combines the quality (i.e., the GI) with the quantity of carbohydrates in a food (GL = GI × available carbohydrates in a serving/100). The GL therefore provides a more accurate picture of the real-life effect of a specific food on postprandial glycaemia [22]. Foods can be classified according to their GL as low (≤10), medium (11–19) or high (≥20) [23].

Observational evidence has shown that diets with high GI and high GL are associated with increased T2DM risk [24], and a causal link has been established between GI and GL and incident T2DM [25]. Two meta-analyses of randomized controlled trials (RCTs) also suggest that low-GI diets improve glycemic control, blood lipids and blood pressure, and contribute to reductions in body mass index (BMI) of patients with type 1 (T1DM) and T2DM [26,27]. However, strong evidence is lacking when it comes to GI and other aspects of cardiometabolic health, such as liver and renal function, pro-inflammatory cytokines related to adipose tissue function and gut microbiota. In this review, we aimed to analyze evidence from clinical trials focusing on the effects of low-GI and low-GL diets on different aspects of health in prediabetes and T2DM, and build a report on all relevant outcomes included in the studies.

## 2. Methods

### 2.1. Search Strategy

Literature searches were conducted on the research databases PubMed and ScienceDirect. The following terms were searched on PubMed, specifically in title and abstract fields: “type 2 diabetes”, “type 2 diabetes mellitus”, “diabetes mellitus type 2” and “glycemic index”, “glycaemic index”, “glycemic ind*”, “glycaemic ind*”, “glycemic load*”, “glycaemic load*”. The search results were narrowed down using additional filters for text availability (full text), article type (clinical study), publication date (2013–2023), species (humans), and language (English, Portuguese). The search on ScienceDirect included the terms “type 2 diabetes”, “type 2 diabetes mellitus”, “diabetes mellitus type 2”, “prediabetes”, “glycemic index”, “glycaemic index”, “glycemic load” and “glycaemic load”. Filters for publication date (2013–2023) and article type (research articles) were applied. The references of relevant papers were scanned through to look for additional papers that could potentially fit the inclusion criteria.

Duplicate papers that emerged during the literature search were identified and excluded. The remaining papers were subjected to two rounds of appraisal to be considered eligible and included in this review. Initially, every article was screened at title and abstract level; then, full-text reviews of articles that passed the first screening were conducted.

### 2.2. Study Selection

This review aimed to include RCTs conducted exclusively on individuals with T2DM or prediabetes, published between 2013 and 2023 and written in English or Portuguese. Inclusion criteria were defined according to the PICO (Population, Intervention, Comparison, Outcome) framework (Table 1). Studies had to include low-GI or low-GL diets and/or foods; the intervention and control groups had to differ in terms of dietary GI and/or GL. Study outcomes pertaining to glycemic control, blood lipids, blood pressure, inflammation, adiposity and CVD were of interest. The length of the studies had to be three weeks or more. 

Exclusion criteria were as follows: participants with T1DM, gestational diabetes; participants without diabetes or prediabetes at baseline; reviews, systematic reviews, meta-analyses, observational, cross-sectional, in vitro and animal studies; uncontrolled trials; study protocols; papers unrelated to GI and GL; papers where the goal was to measure the GI, GL and/or glycemic response (GR) of a certain food or meal; study length shorter than three weeks; studies that did not include outcomes of interest; lack of outcome data at baseline and at the end of the study period; intervention studies designed in a way that made it impossible to isolate the effect of a low-GI or -GL diet.

## 3. Results

### 3.1. Search Results and Study Selection

**Figure 1:**
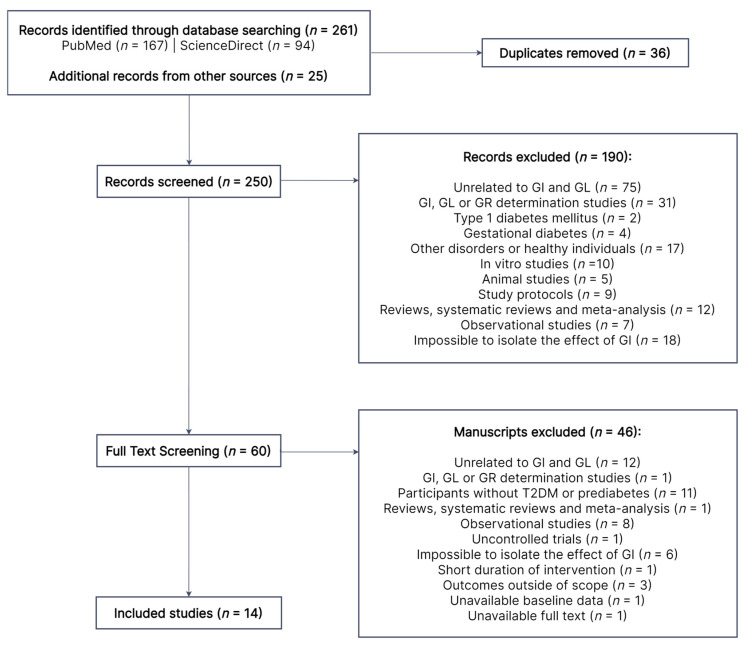
Literature search flow diagram. Out of the initial 250 records, 190 were excluded by title and abstract. A total of 60 papers were subjected to full-text review, 14 of which were eligible. GI, glycemic index; GL, glycemic load; GR, glycemic response; T2DM, type 2 diabetes mellitus.

As shown in Figure 1, 261 records were identified from searches in PubMed and ScienceDirect, in addition to 25 other records obtained from the reference list of relevant papers. After excluding duplicates (*n* = 36), 250 records were screened for title and abstract, 190 of which were excluded for various reasons. The remaining 60 full-text manuscripts were assessed for eligibility. A total of 46 papers were excluded due to the reasons described in Figure 1, while 14 studies were deemed eligible. Three studies were excluded because their study outcomes (satiety [28], cognitive function [29] and episodic memory [30], respectively) were outside of the scope of this review.

### 3.2. Study Characteristics

The main characteristics of the included RCTs [31,32,33,34,35,36,37,38,39,40,41,42,43,44] are presented in Table 2. A total of 1055 individuals with prediabetes or T2DM participated. Studies were published between 2014 and 2022, across 11 countries and 4 continents (America, Asia, Europe and Oceania). The longest intervention was three years long [41], while others ranged from one [35] to six months [33,34,38,40]. Twelve RCTs had a parallel design [31,32,33,34,35,37,38,39,40,41,42,43] and two were crossover studies [36,44]. 

Two trials were conducted exclusively on individuals with prediabetes [35,42] and 11 trials required a T2DM diagnosis [31,32,33,34,36,37,38,39,40,41,43]; one trial included participants with either condition [44]. All studies included individuals of both genders, with 35% [33] to 65% [37] of female participants. However, premenopausal women were excluded in two trials [40,41] and postmenopausal women in one [39]. Pediatric participants were not included in any study. In three studies [32,42,44], researchers exclusively recruited individuals with overweight and obesity, while others also included participants with normal weight [31,34,36,43] or did not impose any restrictions in this regard [33,35,38,39,40,41]. Trials including only prediabetic patients restricted all kinds of hypoglycemic medication [35,42]. One study allowed the participation of individuals who were on metformin [39], while another allowed all types of oral hypoglycemic drugs [38]; other studies excluded participants using acarbose [40] and insulin [32,36,41,43]. Most authors required that the doses of hypoglycemic and other drugs were stable prior and during the intervention [32,33,34,36,37,39,40,41,43] to prevent bias. Alalwan et al. [31], Cai et al. [38] and Mateo-Gallego et al. [44] were the only ones who did not restrict participation based on type or changes in medication. All studies imposed some restrictions with regard to the presence of additional comorbidities. 

Interventions were mostly low-GI [33,34,35,38,39,40,41] or low-GL diets [36]. The remaining studies included interventions with specific low-GI foods [31,42,44], low-GI and low-GL foods [32], and low-GI meals [37,43]. In two studies [32,42], the dietary intervention included energy restriction, i.e., participants followed hypocaloric diets. Table 2 shows further details on each study intervention. 

### 3.3. Glycemic Control

Table 3 portrays the main results of included studies regarding glycemic control and other significant variables. The interventions with low-GI/GL diets, foods or meals had mostly beneficial effects on glycemic control of participants (Table 3). Of the 13 RCTs [31,32,33,34,35,36,37,38,39,41,42,43,44] where glycemic control was assessed, 7 [33,34,35,38,42,43,44] found favorable effects of the low-GI/GL interventions in at least one parameter of glycemic control compared with the control group (intervention vs. control, *p* < 0.05). 

The parameters with the greatest benefit from the low-GI and -GL interventions were glycated hemoglobin (HbA1c) [33,34,38,43,44], homeostatic model assessment for insulin resistance (HOMA-IR) [33,42,44] and fasting insulin [33,37,39,41]. Fasting glucose decreased in the study conducted by Cai et al. [38], while, in other studies, the effect on fasting glucose was similar (*p* > 0.05) between the study groups [32,35,36,37,39,41,42,44]. Argiana et al. [32] found a positive trend in the intervention group, with significant decreases in HbA1c, HOMA-IR, fasting glucose and insulin, but no differences were observed between the studied groups. Gomes et al. [39] observed a similar trend regarding blood fructosamine concentrations, which significantly increased only in the control group. Sipe et al. [35] conducted meal tolerance tests before and after the interventions with low/high-GI diets. The authors found that the low-GI diet reduced the glucose incremental area under the curve (iAUC) and insulin secretion during meal tolerance tests. The effect of the low-GI diet on insulin sensitivity was contradictory—it improved by the Matsuda index, but not by the oral glucose insulin sensitivity (OGIS) index, and total insulin clearance increased. Other measures of β-cell function did not change significantly.

Jenkins [41] observed a trend suggestive of a detrimental effect of the intervention on HbA1c compared with controls. 

### 3.4. Anthropometry, Body Composition and Nutritional Status

Six [32,33,34,39,42,43] out of eleven RCTs [31,32,33,34,36,37,39,41,42,43,44] in which anthropometry, body composition and/or nutritional status were assessed had positive results, i.e., the low-GI/GL interventions were more beneficial for participants compared with the control diets (Table 3) (intervention vs. control, *p* < 0.05). The intervention implemented by Argiana et al. [32] led to decreased hip circumference. Gomes et al. [39] saw a decrease in percentage of fat mass, despite lack of effect on BMI, waist circumference or waist-to–hip ratio. König et al. [42] found decreased body weight and BMI. In the study by Li et al. [43], there were significant reductions in waist circumference and waist–hip ratio in the intervention group; body weight, BMI and fat mass increased in the control group, although differences between study groups were not significant for body weight. In the same study [43], percentage of body water decreased in the control group, whereas Mini Nutritional Assessment (MNA) score did not change throughout the study. Pavithran et al. [34] observed reduced body weight, BMI, triceps skinfold thickness, and total, truncal, android and gynoid fat mass in the intervention group compared with the control; there were no significant effects of this intervention on other parameters, including fat free and lean mass. In their study published in August 2020, Pavithran et al. [33] found decreased body weight and a tendency for decreased waist circumference post-intervention. 

Other studies looking into anthropometric and body composition variables [31,36,37,41,44] did not detect any tendencies or significant differences between groups.

### 3.5. Lipid Profile

The effects of the interventions on lipid profile were mostly comparable to those of the control diets in the nine RCTs [31,32,33,34,36,37,39,41,44] that assessed blood lipids (Table 3). Four RCTs [32,33,37,39] found significant positive effects in the intervention vs. the control group (*p* < 0.05) in at least one parameter of lipid profile. In the case of the study by Gomes et al. [39], they observed a significant and beneficial reduction in non-esterified free fatty acids in the intervention compared with the control group. In three other studies [31,34,44], only positive trends were identified. 

Mateo-Gallego et al. [44] identified a negative trend regarding the effects of the intervention on LDL-C blood concentrations—LDL-C significantly increased in the intervention group, compared with a non-significant increase in the control group. In the study by Jenkins et al. [41], individuals in the control group had an increase in HDL-C and triglycerides, and differences between study groups was significant (*p* < 0.05). 

### 3.6. Blood Pressure

Office blood pressure was measured in five studies [32,33,41,43,44] (Table 3). Argiana and colleagues [32] observed a tendency for decreasing systolic and diastolic blood pressure in both groups. In the study by Li et al. [43], systolic, diastolic and mean arterial pressure all increased (*p* < 0.05) in the control group, but the effect of the intervention was not significant (*p* > 0.05). On the contrary, Mateo-Gallego et al. [44] found a trend for decreased systolic blood pressure in controls (*p* < 0.05) compared with the intervention group (*p* > 0.05). Jenkins [41] and Pavithran et al. [33] did not find significant effects of their interventions on systolic or diastolic blood pressure. However, heart rate increased in the control group in the study by Jenkins et al. [41], with the effect of the control diet (high fiber) being significantly different from that of the low-GI diet. 

### 3.7. CVD Risk

As shown in Table 3, vessel wall volume had increased in both groups by the end of the intervention conducted by Jenkins et al. [41]. However, the increase was significant only in the control group and difference between groups was not significant. There was also no significant changes in Framingham risk score in this study.

Ha et al. [40] quantified the serum concentrations of high sensitivity cardiac troponin I (hs-cTnI) and galectin-3, which are biomarkers of subclinical cardiac injury and fibrosis. While the authors do not present the p value within each group, the effect of the intervention and control diets was comparable for both markers (*p* > 0.05 between groups).

### 3.8. Inflammatory Markers

Significant improvements in biomarkers of inflammation were found in four RCTs [32,33,38,41], specifically in C-reactive protein (CRP) [41], high-sensitivity CRP (hs-CRP) [32,33,38] and interleukins (IL) 6 [38] (Table 3) and 1β [38] (not shown). Gomes et al. [39] detected an increase in tumor necrosis factor α (TNF-α) in the control group and no change in the intervention group. The intervention implemented by Mateo-Gallego and colleagues [44] was the only one that did not lead to reduced inflammatory biomarkers, although only CRP was assessed. Other authors also did not see changes between the intervention and control groups on CRP [39], IL-6 [32] and fibrinogen [39].

Reductions in these inflammatory markers were always associated with improvements in other outcomes, including glycemic control [33,38], reductions in body weight [33], hip circumference [32] and fat mass [39], blood lipids [32,33,39], renal function [41] and intestinal microbiota [38].

### 3.9. Hormones

Argiana et al. [32] and Gomes et al. [39] assessed serum adiponectin concentration, while Argiana et al. [32] also measured serum leptin. These low-GI/GL interventions did not have a significant effect on these parameters compared with controls (Table 3).

### 3.10. Hepatic and Renal Function

Markers of liver function were assessed in three studies, all of which were interventions with low-GI/GL foods [32,44] or meals [43]. Significant differences between study groups were not identified in any parameter of liver function in any study (Table 3). However, Argiana et al. [32] did find significant reductions in blood aspartate aminotransferase (AST) in the control group and in gamma-glutamyl transferase (GGT) in the intervention group. In the study by Li et al. [43], there was a significant increase in total plasma protein only in the intervention group.

Jenkins et al. [41] measured parameters of renal function. Serum urea increased significantly in the intervention and control groups (*p* < 0.05), but no differences were found between groups (*p* > 0.05). Significant differences (*p* < 0.05) between study groups were detected for serum creatinine and glomerular filtration rate (eGFR), due to an increase and a reduction in the control group, respectively.

### 3.11. Intestinal Microbiota

The low-GI diet implemented by Cai et al. [38] led to favorable changes in the intestinal microbiota (*p* < 0.05), when intervention and control groups were compared. It included a decrease in the number of *Enterococcus* and *Escherichia coli* and an increase in *Bifidobacterium* and *Lactobacillus* (Table 3).

### 3.12. Quality of Life

Alalwan and colleagues [31] applied the 36-Item Short Form Survey (SF-36) to their participants to measure quality of life (Table 3). Both the total score and the specific mental health score significantly increased in the intervention group, suggesting an increased quality of life in the participants from the intervention group. Moreover, significant differences were observed between groups (intervention vs. control, *p* < 0.05).

### 3.13. Dietary Intake

Every study besides those by Cai et al. [38], König et al. [42] and Sipe et al. [35] assessed dietary intake (Table 4). The methods used were three to seven-day food records [31,36,37,39,40,41,44] and one to three-day 24 h recalls [32,33,34,43], either alone [37,39,40,41,43,44] or in combination with a food frequency questionnaire (FFQ) [33,34,36], a photo diary of all meals [31] or a diary for sweets consumption [32]. Ward et al. [36] were the only authors to use a validated FFQ and to present the average daily intakes during the intervention, instead of presenting dietary intake at baseline vs. at the end of the study period.

Baseline daily energy intake ranged from 995 ± 201 kcal [36] to 2432 ± 581 kcal [39], whereas the percentage of energy obtained from carbohydrates varied between 38.6% [36] and 64.62 ± 5.56% [33]. At the end of the study, dietary fiber intake ranged from 11.6 ± 3.1 g/day [31] to 35.5 g/day [41]. Participants in the studies by Gomes et al. [39], Ha et al. [40] and Jenkins et al. [41] reduced their daily energy intake from baseline to the end of the study in around 200 to 400 kcal.

Gomes et al. [39], Ha et al. [40] and Jenkins et al. [41] estimated the GI of the diet of each group at baseline and at the end of the study. The average baseline GI was similar between study groups within each study. Baseline GI was classified as medium in the study by Gomes et al. [39] and high in the others [40,41]. The low-GI diets led to reduced GI in the intervention groups of all three studies, but only participants in the Gomes et al. study [39] reached a low GI. The GL was considered high in the three studies, although much lower in the Gomes et al. study [39] compared with the others.

## 4. Discussion

This paper aimed to review the experimental evidence published in the last decade regarding the effects of diets with low GI and low GL on several aspects of health in individuals with prediabetes and T2DM. The selected RCTs included outcomes related to glycemic control, which have been the focus of most of the research on GI and diabetes. However, we included additional, less explored, outcomes including several aspects of cardiometabolic health, nutritional status and quality of life. 

Diet and lifestyle are fundamental aspects of T2DM management that should be optimized to improve glycemic control and treatment outcomes in T2DM. Our results suggest that diets low in GI and GL are a good option for patients with prediabetes and T2DM, with overall benefits in terms of glycemic control and anthropometry. Effects on lipid profile were inconsistent. The effects on other aspects of cardiometabolic health were mostly positive, although few studies documented such parameters.

The interventions with low-GI and/or low-GL foods, meals and diets described in this review exerted overall positive effects on glycemic control—particularly HbA1c, HOMA-IR and fasting plasma insulin. Two systematic reviews and meta-analyses of RCTs conducted on T1DM and T2DM patients [26,27] also found clinically significant improvements in HbA1c. Unlike our results, they found reductions in fasting blood glucose, but not in fasting blood insulin [26,27] or HOMA-IR [26]. The study by Sipe and colleagues [35] observed that a low-GI diet reduced postprandial glucose and insulin secretion in individuals with prediabetes and obesity, but no changes were seen in measures of β-cell function after a 4-week intervention. On the contrary, the authors of a previous study conducted in a similar population over 12 weeks found that a high-GI diet impaired β-cell function, whereas a low-GI diet reduced hyperglycemia and hyperinsulinemia [45]. This suggests that a longer intervention is likely necessary in order to see improvements in pancreatic β-cell function and to prevent or slow progression to T2DM. 

Most studies that showed benefits of low GI on glycemic control also led to reduced body weight, BMI, waist and hip circumferences, waist–hip ratio and body fat [33,34,42,43]. Exceptions were Mateo-Gallego et al. [44], who did not see significant benefits (*p* > 0.05) in anthropometry or body composition, and Cai [38] and Sipe et al. [35], who did not assess these parameters. Interventions with tendencies for an unfavorable effect on glycemic control found similar tendencies regarding anthropometry [37] and lipid profile [41]. Similarly, two meta-analyses found that low-GI diets led to reductions in body weight [27], BMI [26,27] and non-significant reductions in waist circumference [27]. In this review of the literature, improved anthropometric profiles and body composition were seen in interventions with [32,42] and without [33,34,39,43] associated caloric restriction, as well as in normal weight [43], overweight [33,34,39] and obese populations [32,42]. On the other hand, weight loss was only seen in people with obesity in the meta-analysis by Zafar et al. [26].

The two previously mentioned meta-analyses [26,27] saw improvements in lipid profiles associated with low-GI diets, and to a larger extent in prediabetes than in T2DM [26]. Most interventions included in this review, however, did not yield significant benefits in terms of blood lipids. Improvements were documented in three studies in LDL-C [37], HDL-C [32] and triglycerides [33].

The results obtained by Jenkins et al. [41] are particularly relevant, since their intervention was the longest within this review (3 years) and the only one to surpass 6 months. This may therefore provide important insights into the long-term effects of low-GI diets. Jenkins et al. [41] found a very small increase (0.02%) in HbA1c in the low-GI diet group and comparable weight loss at 3 years between the study groups. However, HbA1c levels and body weight were reduced in the intervention group throughout the first 9 and 15 months of the study, respectively. In the per-protocol analysis, reductions were further extended until 15 months, suggesting that better outcomes were expected when participants adhered to the low-GI diet. Despite this and the worsening of HDL-C and TG in the low- vs. high-GI group, participants in the latter had a small increase in vessel wall volume—a quantitative measure of atherosclerosis [46]—and a small decrease in renal function, which did not occur in the low-GI group. This suggests that a low-GI diet may exert beneficial effects on prevention of macro and microvascular complications of diabetes. 

Low-GI diets can lower blood pressure in hypertensive and pre-hypertensive individuals [47], but these effects were negligible in T2DM patients [27]. In this review, most studies that measured blood pressure found improvements in diastolic blood pressure, heart rate and mean arterial pressure, as well as a non-significant decrease in systolic blood pressure.

T2DM and metabolic syndrome are associated with a chronic state of low-grade inflammation, with increased serum levels of CRP and pro-inflammatory cytokines, such as IL-6, IL-1β and TNF-α [48]. High levels of CRP are linked to a higher risk of CVD and mortality in T2DM [48], and there is correlation between hs-CRP and diabetic kidney disease [49]. Our results suggest that eating low-GI foods and diets reduces these inflammatory biomarkers, which may contribute to a lower risk of CVD and other complications of diabetes. The synthesis of TNF-α by adipocytes induces lipolysis and the release of non-esterified free fatty acids into circulation, which triggers insulin resistance [50,51]. In the study by Gomes et al. [39], the high-GI diet led to an increase in serum levels of TNF-α and non-esterified free fatty acids, while the low-GI diet did not. This was likely the result of a decrease in fat mass seen in the low-GI group, which may have affected TNF-α synthesis in the adipose tissue. 

According to our results, low-GI interventions seem to have little impact on serum levels of leptin, adiponectin and liver enzymes. Cai et al. [38], however, found improvements in gut microbiota composition, with decreases in *Enterococcus* and *E. coli* and increased *Bifidobacterium* and *Lactobacillus*. Finally, participants reported improved quality of life and mental health after a 4 month-long intervention with a low-GI food in the study by Alalwan et al. [31]. However, the authors attributed this to the cultural and religious significance of the specific food (dates) consumed in the intervention group rather than its low GI.

To the best of our knowledge, this is the first review on the topic of dietary GI and T2DM to include such a large spectrum of variables. In turn, this approach made it possible to identify areas of knowledge that need further exploring in future studies, such as the impact of low-GI/GL diets on inflammation, liver enzymes and gut microbiota. This review had some important limitations as well, which reflect methodological issues of the included studies, but also general issues of nutritional research and dietary intervention trials. Some of the most notable limitations were the short duration (≤3 months) of most studies [32,35,36,37,39,42,43,44] and the contrasts regarding length of the different interventions. Study samples were often small and/or heterogeneous [32,33,36,39,42,43,44], specifically regarding BMI [39], diabetes duration and presence of additional comorbidities [36]. The fact that many studies allowed participants to take different types of hypoglycemic drugs may also have influenced the results, as these can directly affect the glycemic response to foods and improve glycemic control. However, most authors controlled this bias by requiring that the type and dosage of medication remained stable before and during the intervention. Exceptions were Alalwan [31], Cai [38] and Mateo-Gallego et al. [44], who did not control for this variable. The lack of dietary intake assessment [35,38,42] or the lack of analysis of dietary intake data [32,34,44] was another limitation.

For ethical reasons, a true control diet was not used in some studies. Instead, participants in those control groups were instructed to follow a high fiber diet [38,40,41] or a hypocaloric diet combined with a lifestyle intervention [42], which may have masked some of the effects of the low-GI/GL interventions. 

Some authors reported low participant adherence and significant dropout rates [33,41,43]. Additionally, compliance was assessed by self-report in the Argiana et al. [32] and Jenkins et al. [41] studies. Implementing strategies, especially within long-term trials, to increase adherence is crucial [52], such as providing study foods to participants [53]. On the other hand, this can mask the applicability of the study to real-life settings [41], since any potential benefits of low-GI diets become irrelevant if individuals cannot adhere to them. Therefore, the goal with any dietary intervention to manage chronic illnesses such as T2DM should be to find a healthy diet, and one that the patient finds pleasurable and is able to adhere to it in the long term.

## 5. Conclusions

Adopting low-GI and low-GL diets as well as including low-GI/GL foods on a daily basis may be a good dietary approach for patients with prediabetes and T2DM. The results of our literature review have shown that low-GI and -GL interventions have clear benefits in terms of short-term glycemic control, and can aid in weight loss and adiposity reduction. However, it is uncertain whether these benefits persist over the long term compared to other dietary patterns. This type of intervention may have important benefits that add to the effects of hypoglycemic drugs. On the other hand, preferring low- over high-GI/GL foods may contribute to a slower progression of the condition and retard common diabetes health complications.

The interventions and study samples differed greatly between studies, which compromised comparability of the results. No recommendations can be made at this time regarding the effects of low GI/GL on outcomes such as those related to hormone secretion, pro-inflammatory cytokines and gut microbiota, as very few studies reported these results.

Conducting longer trials is the only way to properly address these unanswered questions. Future trials should also address outcomes that have been less explored to date, which could provide further insights into the metabolic effects of low-GI diets on individuals with diabetes. Finally, more data are necessary regarding the effect of low-GI and -GL diets on CVD events and mortality in T2DM. 

## Figures and Tables

**Table 1:** Selection criteria for studies using the PICO framework.

**Population**	Individuals with T2DM or prediabetes
**Intervention**	Low-GI/GL food, meal or diet
**Comparison**	Higher-GI/GL food, meal or diet
**Outcomes**	Outcomes related to glycemic control, blood lipids, blood pressure, inflammation, adiposity, CVD and other outcomes considered useful
**Study length**	≥3 weeks

CVD, cardiovascular disease; GI, glycemic index; GL, glycemic load; PICO, population, intervention/exposure, comparison and outcomes; T2DM, type 2 diabetes mellitus.

**Table 2:** Characteristics of study design and study populations at baseline.

Study	Country (Year)	Study Design	Type of Intervention	Study Length	Diagnosis	DM Medication	Gender (%F)	Group	*n*	Age (Years)	BMI (kg/m^2^)	HbA1c (%)	Intervention Description
[31]	Bahrain (2020)	Parallel	Low-GI food	4 mo	T2DM	-	61	T	100	20–65	>22	6–10	
								I	50	55.3 ± 2.7	28.5 ± 7.7	6.6 ± 0.8	3 dates consumed at breakfast
								C	50	56.9 ± 4.4	29.9 ± 4.1	6.6 ± 0.7	No date consumption
[32]	Greece (2015)	Parallel	Low-GI/GL food	3 mo	T2DM	No insulin	53	T	58	40–65	25–40	<8	
								I	28	61.3 ± 1.4	32.7 ± 0.8	6.6 ± 0.1	Hypocaloric diet + 4 weekly portions of low-GI/GL sweets
								C	30	63.0 ± 1.3	32.4 ± 0.8	6.8 ± 0.2	Hypocaloric diet + 1 weekly portion of favorite sweet
[37]	Thailand (2018)	Parallel	Low-GI meal	3 mo	T2DM	Any type	65	T	110	≥18	-	7–9	
								I	53	56.0 ± 8.9	27.9 ± 4.2	7.9 ± 0.7	Low-GI meal replacement once per day with controlled diets
								C	57	56.3 ± 10.0	27.7 ± 4.9	7.8 ± 0.6	Controlled diets
[38]	China (2017)	Parallel	Low-GI diet	6 mo	T2DM	Oral medication	46	T	130	-	-	-	
								I	65	56.9 ± 3.9	-	-	Low-GI high-fiber diet + exercise
								C	65	56.4 ± 3.7	-	-	High fiber diet + exercise
[39]	Brazil (2017)	Parallel	Low-GI diet	1 mo	T2DM	Metformin	50	T	20	18–55	-	-	
								I	10	44.3 ± 4.8	28.8 (22.5–33.9)	-	Low-GI diet + 2 daily low-GI test meals consumed in the lab
								C	10	41.1 ± 3.2	28.6 (25.4–37.5)	-	High-GI diet + 2 daily high-GI test meals consumed in the lab
[40]	Canada (2017)	Parallel	Low-GI diet	6 mo	T2DM	No acarbose	39	T	201	-	-	6.5–8	
								I	102	60.2 ± 9.5	30.5 ± 6.1	7.2 ± 0.6	Low-GI high-fiber diet
								C	99	61.3 ± 8.7	31.0 ± 5.5	7.1 ± 0.5	High-cereal fiber diet
[41]	Canada (2022)	Parallel	Low-GI diet	3 y	T2DM	No insulin	39	T	169	-	-	6.5–8	
								I	86	61 ± 9	30 ± 5	7.1 ± 0.6	Low-GI diet
								C	83	62 ± 6	29 ± 5	7.1 ± 0.5	Wheat-fiber diet
[42]	Germany (2014)	Parallel	Low-GI food	6 wk	Pre-DM	None	62	T	42	54 ± 8	≥25	-	
								I	28	-	32.9 ± 3.2	-	Hypocaloric diet + 2 daily low-GI meal replacements
								C	14	-	32.8 ± 2.3	-	Hypocaloric diet + healthy lifestyle
[43]	China (2014)	Parallel	Low-GI meal	3 mo	T2DM	No insulin	39	T	54	18–75	18.5–35	-	
								I	36	56.7 ± 8.6	24.6 ± 2.6	6.7 ± 0.9	Breakfast replaced with a low-GI, multi-nutrient supplement
								C	18	54.5 ± 10.1	23.7 ± 2.9	6.5 ± 0.6	Healthy breakfast
[44]	Spain (2020)	Crossover	Low-GI food	10 wk	T2DM Pre-DM	-	38	T	43	55.8 ± 7.4	31.9 ± 3.1	6.0 ± 0.6	
								I	42	-	-	-	66 mL/day of alcohol-free beer with modified CHO content
								C	43	-	-	-	66 mL/day of regular alcohol-free beer
[34]	India (2020)	Parallel	Low-GI diet	6 mo	T2DM	Any type	42	T	36	35–65	≤35	7–10	
								I	18	52 ± 7.7	26.8 ± 5.0	8.3 ± 0.9	Kerala cuisine low-GI diet
								C	18	-	27.3 ± 2.7	8.2 ± 1.0	Usual diet
[33]	India (2020)	Parallel	Low-GI diet	6 mo	T2DM	Any type	35	T	80	35–65	-	7–10	
								I	40	54.4 ± 7.6	26.4 ± 3.0	8.4 ± 1.0	Kerala cuisine low-GI diet
								C	40	51.9 ± 7.4	26.8 ± 3.3	8.3 ± 1.0	Usual diet
[35]	USA (2022)	Parallel	Low-GI diet	1 mo	Pre-DM	None	49	T	35	18–65	-	-	
								I	17	58.1 ± 1.5	32.5 ± 1.2	5.8 ± 0.1	Low-GI diet
								C	18	50.6 ± 2.4	32.4 ± 1.5	5.8 ± 0.1	High-GI diet
[36]	Australia (2020)	Crossover	Low-GL diet	2 mo	T2DM	No insulin	36	T	17	58.0 ± 6.6	29.9 ± 3.5	7.0 ± 0.9	
								I	17	-	-	-	20% daily energy intake replaced with lupin-enriched foods
								C	17	-	-	-	20% daily energy intake replaced with wheat-based control foods

BMI, body mass index; C, control group; CHO, carbohydrate; DM, diabetes mellitus; F, female; GI, glycemic index; GL, glycemic load; HbA1c; glycated hemoglobin; I, intervention group; mo, month; pre-DM, prediabetes; T, total sample; T2DM, type 2 diabetes mellitus; wk, week.

**Table 3:** Summary of the major results reported in the literature, for both studied groups and for the evaluation between intervention and control group.

Study Reference		[31]	[32]	[37]	[38]	[39]	[40]	[41]	[42]	[43]	[44]	[34]	[33]	[35]	[36]
**Glycemic control**															
HbA1c	I		*		•			*		*		*	*		
	C			*	•					*		•			
	*p*	>0.05	>0.05	>0.05	**<0.05**			>0.05		**<0.05**	**<0.05**	**<0.05**	**<0.05**		
HOMA-IR	I		*						*		*		*		
	C								*						
	*p*		>0.05						**<0.05**		**<0.05**		**<0.05**		
Fasting glucose	I		*		•			*	*						•
	C				•			*	*						•
	*p*		>0.05	>0.05	**<0.05**	>0.05		>0.05	>0.05		>0.05			>0.05	>0.05
Fasting insulin	I		*		•				*		*		*		•
	C				•										•
	*p*		>0.05		**<0.05**				**<0.05**		**<0.05**		**<0.05**		>0.05
C-peptide	I														
	C														
	*p*														>0.05
Fructosamine	I														
	C					*									
	*p*					**>0.05**									
Glucose sensitivity	I														
	C														
	*p*													>0.05	
**Anthropometry**															
Body weight	I		*	*				*	*		*	*	*		•
	C		*	*				*	*	*	*				•
	*p*		>0.05	>0.05				>0.05	**<0.05**	>0.05	>0.05	**<0.05**	**<0.05**		>0.05
BMI	I		*	*				*	*			•			
	C		*	*				*	*	*		•			
	*p*	>0.05	>0.05	>0.05		>0.05		>0.05	**<0.05**	**<0.05**		**<0.05**			
Waist circumference	I		*							*	*		*		
	C		*								*				
	*p*		>0.05	>0.05		>0.05				**<0.05**	>0.05	>0.05	>0.05		
Hip circumference	I		*												
	C														
	*p*		**<0.05**							>0.05					
Waist–hip ratio	I									*					
	C														
	*p*		>0.05			>0.05				**<0.05**					
Triceps skinfold	I											*			
	C														
	*p*											**<0.05**			
**Body composition**															
Fat mass	I					*						•			
	C									*		•			
	*p*					**<0.05**				**<0.05**	-	**<0.05**			
Fat free mass	I											•			
	C											•			
	*p*										-	>0.05			
**Lipid profile**															
TC	I	*						*				*	*		•
	C							*				•	*		•
	*p*	>0.05	>0.05	>0.05		>0.05		>0.05			-	>0.05	>0.05		>0.05
LDL-C	I										*	•	*		•
	C							*				•			•
	*p*	>0.05	>0.05	**<0.05**				>0.05			>0.05	>0.05			>0.05
HDL-C	I										*				•
	C		*	*				*							•
	*p*	>0.05	**<0.05**	>0.05		>0.05		**<0.05**			>0.05	>0.05	>0.05		>0.05
VLDL	I											*			
	C											•			
	*p*											>0.05	>0.05		
Triglycerides	I											*	*		•
	C							*				•	*		•
	*p*	>0.05	>0.05	>0.05		>0.05		**<0.05**			-	>0.05	**<0.05**		>0.05
LDL-C/HDL-C	I														
	C							*							
	*p*		>0.05					>0.05							
ApoB	I												*		
	C												*		
	*p*										-		<0.05		
**Blood pressure**															
Systolic blood pressure	I		*												
	C									*	*				
	*p*		>0.05					>0.05		>0.05	>0.05		>0.05		
Diastolic blood pressure	I		*					*			*				
	C							*		*	*				
	*p*		>0.05					>0.05		**<0.05**	>0.05		>0.05		
**CVD risk**															
Vessel wall volume	I														
	C							*							
	*p*							>0.05							
Framingham risk score	I														
	C														
	*p*							>0.05							
Hs-cTnl	I						•								
	C						•								
	*p*						>0.05								
Galectin-3	I						•								
	C						•								
	*p*						>0.05								
**Inflammatory markers**															
C-reactive protein	I							*							
	C														
	*p*					>0.05		**<0.05**			-				
High sensitivity C-reactive protein	I		*		•								*		
	C				•										
	*p*		**<0.05**		**<0.05**								**<0.05**		
IL-6	I				•										
	C				•										
	*p*		>0.05		**<0.05**	-									
**Hormones**															
Leptin	I														
	C														
	*p*		>0.05												
Adiponectin	I														
	C														
	*p*		>0.05			>0.05									
**Hepatic function**															
AST	I														
	C		*												
	*p*		>0.05								-				
ALT	I														
	C														
	*p*		>0.05								-				
GGT	I		*												
	C														
	*p*		>0.05								-				
Uric acid	I														
	C														
	*p*		>0.05								-				
**Renal function**															
Urea	I							*							
	C							*							
	*p*							>0.05							
Creatinine	I														
	C							*							
	*p*							**<0.05**							
eGFR	I														
	C							*							
	*p*							**<0.05**							
**Intestinal microbiota**															
*Enterococcus*	I				•										
	C				•										
	*p*				**<0.05**										
*Escherichia coli*	I				•										
	C				•										
	*p*				**<0.05**										
*Bifidobacterium*	I				•										
	C				•										
	*p*				**<0.05**										
*Lactobacillus*	I				•										
	C				•										
	*p*				**<0.05**										
**Quality of life**															
Total SF-36 score	I	*													
	C														
	*p*	**<0.05**													
SF-36 mental health	I	*													
	C														
	*p*	**<0.05**													
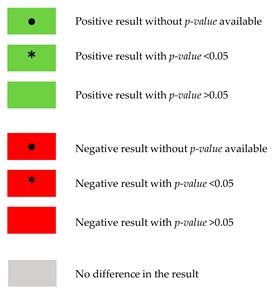															

ALT, alanine aminotransferase; apoB, apolipoprotein B; AST, aspartate aminotransferase; BMI, body mass index; C, control group; CVD, cardiovascular disease; eGFR, estimated glomerular filtration rate; GGT, gamma-glutamyl transferase; HbA1c, glycated hemoglobin A1c; HDL-C, high-density lipoprotein cholesterol; HOMA-IR, homeostatic model assessment for insulin resistance; hs-cTnI, high sensitivity cardiac troponin I; I, intervention group; IL-6, interleukin-6; LDL-C, low-density lipoprotein cholesterol; SF-36, 36-Item Short Form Survey; TC, total cholesterol; VLDL, very-low-density lipoprotein.

**Table 4:** Dietary intake assessment methods, daily energy and nutrients intake, diet GI and GL results of the included studies.

Study	Dietary Intake Assessment		Group	Energy (kcal/Day)		CHO (E%)		Dietary Fiber (g/Day)		Diet GI		Diet GL	
				Baseline	End	Baseline	End	Baseline	End	Baseline	End	Baseline	End
[31]	Food record	5 days	I	2233 ± 61	2230 ± 65	48.9	47.1	14.6 ± 3.1	11.6 ± 3.1	-	-	-	-
	Photo diary of meals		C	2216 ± 55	2230 ± 65	64.4	52.5	14.3 ± 4.9	16.7 ± 3.1	-	-	-	-
[32]	24 h recall	1 day		-	-	-	-	-	-	-	-	-	-
	Diary of sweets consumption												
[37]	Food record	-	I	1350 ± 310	-	50	-	-	-	-	-	-	-
			C	1210 ± 3730	-	50	-	-	-	-	-	-	-
[39]	Food record	3 days	I	2218 ± 602	1998 ± 596	59.8 ± 9.3	57.0 ± 8.1	19.6 ± 7.6	21.4 ± 7.2	63 ± 6	54 ± 4	39.3 ± 12.4	32.5 ± 10.6
			C	2432 ± 581	2013 ± 591	53.5 ± 8.4	57.9 ± 7.7	18.5 ± 5.4	20.6 ± 6.1	66 ± 4	72 ± 3	36.2 ± 10.1	39.3 ± 12.4
[40]	Food record	7 days	I	1916 (1805–2026)	1706 (1607–1805)	42.2 (40.9–43.4)	44.0 (42.4–45.6)	26.6	31.9	80.8 (79.6–82.0)	69.6 (67.7–71.4)	161.6 (151.8–171.4)	128.9 (120.5–137.3)
			C	1830 (1720–1940)	1690 (1594–1786)	45.4 (43.7–47.0)	47.5 (45.8–49.1)	25.8	26.5	81.5 (80.4–82.7)	83.5 (82.4–84.7)	169.0 (156.5–181.5)	166.0 (155.5–176.4)
[41]	Food record	7 days	I	1757 ± 489	1529 ± 438	48.0 ± 8.1	49.2 ± 7.9	26.9	35.5	78.9 ± 5.6	67.3 ± 7.5	102 ± 32	73 ± 26
			C	1765 ± 415	1533 ± 424	47.7 ± 8.0	49.3 ± 8.1	25.6	28.1	79.5 ± 6.9	81.3 ± 5.6	103 ± 29	93 ± 31
[43]	24 h recall	3 days	I	1567 ± 513	1446 ± 401	54.6 ± 8.1	45.6 ± 8.8	-	-	-	-	-	-
			C	1426 ± 353	1523 ± 550	52.1 ± 9.4	42.7 ± 8.0	-	-	-	-	-	-
[44]	Food record	3 days		-	-	-	-	-	-	-	-	-	-
[34]	24 h recall *	1 day		-	-	-	-	-	-	-	-	-	-
	FFQ	59 items											
[33]	24 h recall	1 day	I	1430 ± 182	1511 ± 138	64.6 ± 5.6	61.6	-	-	-	-	-	-
	FFQ	60 items	C	1555 ± 233	1450 ± 157	63.3 ± 6.0	65.7	-	-	-	-	-	-
[36]	Food record	7 days	I	995 ± 201	-	38.6	-	29 ± 6	-	-	-	-	-
	FFQ **	-	C	1117 ± 380	-	50.1	-	22 ± 6	-	-	-	-	-

C, control group; CHO, carbohydrate; E%, percentage of energy intake; FFQ, food frequency questionnaire; GI, glycemic index; GL, glycemic load; I, intervention group. * Baseline data not available. ** Validated tool.
